# Cerebro-Spinal Fluid in General Paralysis

**Published:** 1903-10-03

**Authors:** 


### CEREBRO-SPINAL FLUID IN GENERAL PARALYSIS.

About a year ago Joffroy and Mercier[1] drew attention to the fact that in general paralysis there is an augmentation of the number of white blood corpuscles in the cerebro-spinal fluid. They found that the phenomenon was almost always present in cases of general paralysis, and they came to the conclusion that the sign was a valuable aid to diagnosis in this disease. Increase of the number of leucocytes in the cerebro-spinal fluid, that is, above 2 per c.m., was also found in four cases of tabes dorsalis and in one case of syphilitic disease of the spinal cord. From 120 punctures made upon 91 patients the results were so constant that Joffroy and Mercier considered that general paralysis could be excluded if there was absence of leucocytosis in the cerebro-spinal fluid, and conversely that if there was leucocytosis general paralysis might be diagnosed provided that tabes dorsalis could be excluded. Dr. Jost D. Kramer[2] has made further observations in the same direction. He examined 29 patients in all. Eleven of these had general paralysis, and the leucocyte count of the cerebro-spinal fluid showed an excess of leucocytes in every instance, the number per c.m. varying from six to as many as 145 in one case. The remaining 18 patients were suffering from various kinds of mental disease, including dementia præcox, Huntingdon's chorea, epileptic insanity, melancholia, chronic alcoholism, and pseudo-diabetic tabes. In none of them did the leucocytes exceed 2 per c.m. Dr. Kramer prefers to perform the lumbar puncture with the patient sitting upon a chair, or else with the patient lying on his side with the knees strongly flexed. The needle is inserted into the interspace between the fourth and fifth lumbar vertebræ a little to the right of the middle line. Dr. Kramer says that the patient shows very little discomfort at the time of the operation, and no serious symptoms have followed. The necessary precautions against sepsis were, of course, strictly observed.

[1] Journ. of Mental Pathology, Oct.-Nov., 1902. [2] American Journal of Insanity, July, 1903.

